# Antimicrobial Biomasses from Lactic Acid Fermentation of Black Soldier Fly Prepupae and Related By-Products

**DOI:** 10.3390/microorganisms8111785

**Published:** 2020-11-14

**Authors:** Jasmine Hadj Saadoun, Anna Valentina Luparelli, Augusta Caligiani, Laura Ioana Macavei, Lara Maistrello, Erasmo Neviani, Gianni Galaverna, Stefano Sforza, Camilla Lazzi

**Affiliations:** 1 Department of Food and Drug, University of Parma, Parco Area delle Scienze 49/a, 43124 Parma, Italy; jasmine.hadjsaadoun@unipr.it (J.H.S.); annavalentina.luparelli@unipr.it (A.V.L.); erasmo.neviani@unipr.it (E.N.); gianni.galaverna@unipr.it (G.G.); stefano.sforza@unipr.it (S.S.); 2 Centre BIOGEST-SITEIA, University of Modena and Reggio Emilia, Piazzale Europa 1, 42124 Reggio Emilia, Italy; lauraioana.macavei@unimore.it (L.I.M.); lara.maistrello@unimore.it (L.M.); 3 Department of Life Science, University of Modena and Reggio Emilia, Via Amendola 2, 42122 Reggio Emilia, Italy

**Keywords:** Black soldier flies 1, insects waste 2, puparia 3, antimicrobial 4, fermentation 5, lactic acid bacteria 6, chemical composition 7

## Abstract

Worldwide, thousands of insect species are consumed as food or are used as feed ingredients. *Hermetia illucens*, ‘black soldier fly’, is one of them, and a large amount of puparia and dead adults flies are accumulated during rearing. These materials represent important wastes but no studies are still present in the literature regarding their functional properties and potential reuse. Lactic acid bacteria (LAB) are a heterogeneous group of bacteria contributing to various industrial applications, ranging from food fermentation, chemicals production to pharmaceuticals manufacturing. A LAB feature of industrial interest is their ability to produce antimicrobial metabolites. Considering the scientific and commercial interest in discovering novel antimicrobials, this work will be direct towards fermentation of insect-derived biomasses: puparia and adults insect at the end of life cycle. To the best of our knowledge, the in vitro antimicrobial activity of fermented insects is tested for the first time. This study aimed also to evaluate differences in the composition between fermented and unfermented insects, and to study whether the fermentation and the type of LAB used played a crucial role in modifying the composition of the substrate. Results firstly highlighted fermentability of this species of insects, showed that fermented black soldier flies puparium possess a high antimicrobial activity against tested pathogens. Moreover, result of chemical composition showed that fermented biomass had a higher percentage of fat and a more complex fatty acids profile.

## 1. Introduction

The world population is predicted to increase from 5.4 billion to about 9 billion within a few decades, and it is relevant to find new sources of high quality protein, different from soybean or animal source, to feed this additional population [1].

To accommodate this number, insects represent a good alternative in terms of protein quality and environmental sustainability [2]. They grow and reproduce easily, have low feed conversion rate compared to conventional livestock animals (one kg of insect biomass can be produced from 1.7 Kg of feed biomass, depending on species) and can be reared on agro-industrial by-products [3,4]. In addition to serving as feed, insects provide a high nutritional profile for humans, although the presence of allergens represents a potential hazards that must be further investigated [5,6]. Overall, it is demonstrated that insects production involves low energy, land area utilization and environmental footprints [7].

This study focused on *Hermetia illucens*, also called black soldier flies (BSF), a Diptera belonging to Stratyiomidae family. In accordance with Commission Regulation (EU) 2017/893 [8], this insect is one of the seven species approved for feeding of aquaculture animals and thanks to its high nutritional value [9], and to its ability to convert industrial waste [10,11,12,13,14] is one of the most promising to be used in the feed industry.

Its life cycle includes six larval stages concluding with the prepupa stage. At this point, BSF moves away from the growth substrate and looks for a dry and safe place where it is possible to pupate (pupa stage), performing the metamorphosis and emerging as an adult [15].

Currently, BSF larvae are reared for aquaculture in USA [9], for pet feed in Germany [16] and are included in the list of insect species with the greatest potential to be used also as food with restriction, for all of these cases, on the substrates fed to BSF that must contain products of non-animal origin [8,17].

As a result, there has been an increase in BSF rearing at industrial scale production in the last years [18]. In an industrial mass rearing of BSF, beside frass, several other wastes are generated during the development of the insect. Among these, there are the puparia, which are the shells left after the adult emergence, and the bodies of the dead adults after mating and egg laying. Nowadays, these materials represent a significant waste and no studies are present in literature regarding their functional properties and potential reuse.

In the last decades, several studies reported that one possible strategy to add value to waste material are fermentation processes. Lactic acid bacteria (LAB), a heterogeneous group with recognition as Qualified Presumption of Safety (QPS)-status by EFSA, are the most important microorganisms associated with fermentation and thus exploited for industrial bioprocesses. Recently, the exploitation of LAB strains to valorise by-products and waste into high value added products has been described but their potential on bio-transformations is still an untapped biotechnology resource [19,20,21]. Different metabolites of industrial interest, such as bioactive molecules, can be produced by lactic acid fermentation starting from low-cost substrates. For instance, LAB have a wide range of antimicrobial effects against many pathogens due to the production of organic acids, hydrogen peroxide, and bacteriocins and their ability to convert agro-industrial leftovers into antimicrobial compounds has been recently reported [22,23]. Furthermore, due to these characteristics, fermentation could also be useful to extend shelf life and improve the taste of insect [24]. The metabolic potential of LAB has been studied in different substrates and insects represent a new resource to be explored to produce high-value biochemical molecules.

In this study, we describe the fermentation of BSF and BSF-derived biomasses: prepupae, puparia and adults insects at the end of life cycle were used as substrates for LAB growth and the antimicrobial activity against different pathogens was determined by challenge test. Differences in the chemical composition of BSF biomasses before and after fermentation, in terms of gross composition, lipid and protein profiles, were discussed. To the best of our knowledge, this is the first study focused on biovalorisation of BSF and this type of waste.

## 2. Materials and Methods

### 2.1. Collection of Insect Waste

The BSF prepupae, puparia and dead adult flies used for all the experiments were obtained from the Laboratory of Applied Entomology—BIOGEST-SITEIA, Department of Life Science, University of Modena and Reggio Emilia, Reggio Emilia (RE), Italy.

The BSF material was obtained from a colony initiated in 2016, from larvae purchased from CIMI srl (Cuneo, Italy (CN)). The larvae were reared at 27 °C and 70% relative humidity and fed *at libitum* with Gainesville Housefly diet [25,26]. The prepupae were collected and placed in a dry and ventilated polyethylene container for flies emergence. The newly emerged adults were further transferred in BugDorm^®^ (BD4S3030, MegaView Science Co., Taiwan) provided with sugar cubes and water for adult nutrition and a patent pending device [27] as oviposition site for eggs laid by the BSF females. The colony maintenance was performed three times per week, when the eggs were collected and placed directly on the rearing substrate for hatching of the new larvae, whereas the puparia and dead flies were collected and kept in falcon vials at −20 °C.

### 2.2. Bacterial Strains

Two bacterial strains, *Lacticaseibacillus rhamnosus* 1473 isolated from Parmigiano Reggiano cheese, and *Lactiplantibacillus plantarum* 285, isolated from Brazilian cheese, were used for fermentation. The antimicrobial activity of insect waste was tested toward 3 pathogenic strains belonging to: *Salmonella enterica* (serotype Rissen)*, Listeria monocytogenes* (LMG 21264) and *Escherichia coli* (k88 isolated from piglet’s gut). All the strains belonged to the collection of the Department of Food and Drug (University of Parma, Parma, Italy).

Lactobacilli were maintained at −80 °C in De Man, Rogosa, and Sharpe (MRS) broth (Oxoid, Basingstoke, UK) supplemented with 12.5% glycerol (*v*/*v*). Three pathogenic strains were maintained at −80 °C in tryptic soy broth (TSB) (VWR, Leuven, BE) supplemented with 12.5% glycerol (*v*/*v*).

### 2.3. Fermentation

Puparia and adults were smashed in small particles using IKA A10 laboratory grinder (IKA Werke GmbH e Co., Staufen, Germany), water (70% *v*/*w*) and sugar (8.5% *w*/*w*) were added, then sterilized in autoclave at 121° for 20 min in a glass jar. Prepupae were smashed in small particles using IKA A10 laboratory grinder, added of sugar (8.5% *w*/*w*) and sterilized in autoclave at 121 °C for 20 min in a glass jar. Before fermentation, LAB strains were transferred twice in MRS broth (3% *v*/*v*) and incubated for 24 h at 30 °C for *L. plantarum* 285 and 37 °C for *L. rhamnosus*1473. Afterwards, MRS broth was inoculated (3% *v*/*v*) with each revitalized strain and incubated for 15 h at the specific temperatures of each species, in order to obtain a cell concentration of 9 Log CFU/mL. Each grown bacterial culture was centrifuged (12,857× *g*, 10 min, 4 °C), washed twice in Ringer solution (VWR, UK), and suspended in sterile bidistilled water. 30 g of puparia, adults and prepupae were inoculated individually with each bacterial suspension in order to obtain a final concentration of 7 Log CFU/g, in triplicate. The inoculated substrates were then incubated for 72 h at 30 °C for *L. plantarum* 285 and at 37 °C for *L. rhamnosus* 1473. For microbial counts, 5 g of each sample were homogenized in 45 mL of Ringer solution for 60 s in a Stomacher 400 Circulator (Seaward, England), and serial 10-fold dilutions were performed. Total viable count of the starter was determined using plate count agar on MRS.

### 2.4. Proximate Composition

Standard procedures [28] were used to test moisture, lipid and ash composition of the samples, grinded for 2 min with IKA A10 laboratory grinder before each analysis. Moisture was determined in oven at 105 °C for 24 h. An automatized Soxhlet extractor (SER 148/3 VELP SCIENTIFICA, UsmateVelate, Italy) was used to determinate crude fat, extracted using diethyl ether as solvent. Total ash was determined after mineralization at 550 °C for a total time of 10 h (5 h + 5 h). Total protein content of the samples was calculated from the sum of the amounts of amino acids, determined as in paragraph 2.5. In order to determine the correct protein amounts, the mmoles of each amino acid were multiplied for their residual molecular mass (molecular mass of free amino acid subtracted of the molecular mass of water). Regarding total chitin content of fermented sample, it was estimated as the sum of free glucosamine released after acid hydrolysis as described in the Section 2.6.

### 2.5. Total Amino Acid Profile

Total amino acids determination was carried out as reported by the method described by Leni et al. [29]. All samples (500 mg) were hydrolysed with 6 mL of HCl 6 N at 110 °C for 23 h. At the end of hydrolysis, 7.5 mL of 5 mM Norleucine in HCL 0.1 N, used as internal standard, was added. After filtration, the sample was brought up to volume of 250 mL. Only for the analysis of cysteine, determined as cysteic acid, the acid hydrolysis described above was preceded by a performic acid oxidation. In this case, 2 mL of performic acid freshly prepared (by mixing formic acid with hydrogen peroxide in 9:1 proportion) was added to an amount of 0.5 g sample and kept in an ice bath for 16 h at 0 °C. Then the bromine formed after the addition of 0.3 mL of hydrobromidric acid was removed under nitrogen flow. The hydrolysed samples were analysed by UPLC/ESI-MS, after derivatization with reconstituted AccQ Tag reagent (Waters Co., Milford, USA) according to the method described by Leni et al. (Leni et al., 2020a). Calibration was performed with standard solution prepared mixing 133.3 µL of Norleucine (5 mM), 133.3 µL of amino acids hydrolysate standard mixture (2.5 mM), 133.3 µL of cysteic acid in HCL 0.1 N (2.5 mM) and 100 µL of deionized water.

#### Tryptophan Determination by UPLC/ESI-MS after Alkaline Hydrolysis

Tryptophan analysis was carried out according to the method described by Caligiani et al. [30] with some modifications. 3 mL of 4 N NaOH and 150 µL of 5-methyl- DL-tryptophan standard solution (prepared by mixing 16 milligrams in 100 mL of distilled water) were added to 0.2 hundred milligrams of sample and hydrolysed at 110 °C for 18 h. After alkaline hydrolysis, the solution was neutralized by adding 37% HCl and brought to 25 mL with sodium borate buffer (0.1 M, pH 9.0). The samples were centrifuged for 5 min at 4000 rpm at 4 °C. 0.45 μm nylon filter membrane were used to filter the surnatants collected after centrifugation. UPLC/ESI-MS analysis was performed by using an ACQUITY UPLC separation system with an Acquity BEH C18 column (1.7 µm, 2.1 × 150 mm). The mobile phase was composed by H2O + 0.2% CH3CN + 0.1% HCOOH (eluent A) and CH3CN + 0.1% HCOOH (eluent B). Gradient elution was performed: isocratic 100% A for 1.8 min, from 100% A to 50% A by linear gradient in 11.4 min and 0.8 min at 50% A plus washing step at 0% A (100% B) and reconditioning. Flow rate was set at 0.25 mL/min, injection volume 2 μL, column temperature 35 °C and sample temperature 23 °C. Detection was performed by using Waters SQ mass spectrometer: ESI source in positive ionization mode, capillary voltage 3.2 kV, cone voltage 30 V, source temperature 150 °C, desolvation temperature 300 °C, cone gas flow (N2): 100 L/h, desolvation gas flow (N2): 650 L/h, full scan acquisition (100–2000 m/z), scan duration 1 s.

### 2.6. Determination of Chitin

The total amount of chitin was determined by using a UPLC/ESI-MS quantification of glucosamine after samples acidic hydrolysis, as described by D’Hondt et al. [31] with some modifications. Practically, samples preparation was the same used for total amino acids determination (Section 2.5): 500 mg of BSF puparia, dead adults and prepupae were hydrolysed in 6 N HCl for 23 h at 110 °C, added to 7.5 mL of 5 mM Norleucine as internal standard, filtered and brought to 250 mL. The same procedure was applied to a chitin standard (Sigma Aldrich) to calculate the recovery. UPLC/ESI-MS Analysis conditions were the same described for tryptophan determination.

### 2.7. Determination of Fatty Acids Profile by GC-MS

The determination of fatty acids profile was carried out on the crude fat extracted using the Soxhlet extraction technique (Section 2.4). Before the analysis in GC-MS, acidic-catalysed transmethylations was carried out on 50 mg of BSF fat residue according to the method used by Caligiani et al. [30] with some modifications. Weighed fat of each BSF samples was dissolved in 1 mL of 5% HCl in methanol. The reaction was carried out in oven at 70 °C for 45 min. After cooling, 50 µL of methyl tetracosanoate, used as internal standard, and 2.5 mL of hexane were added. The superior hexane phase containing the fatty acid methyl esters formed during the acid transmethylation was collected and stored. Before the instrumental analysis, a dilution of each extract was performed by adding different quantity of hexane to match the linearity range of the GC-MS instrument. The solutions were split injected (1 µL) on a Thermo Scientific Trace 1300 gas-chromatograph (Thermo Scientific, Waltham, MA, USA) carrying a Supelcowax ms capillary column (30 m, i.d 25 mm, Supelco, Bellafonte, USA) coupled to a Thermo Scientific Trace ISQ mass spectrometer (Thermo Scientific, Waltham, MA, USA). Carrier gas was helium (1 mL/min), injector and detector temperatures were kept at 250 °C, while oven temperature was programmed from 80 to 240 °C at 20 °C/min. Content of each single fatty acid was calculated in relation to the concentration of the internal standard, after calculating the response factors using the Supelco^®^ 37 Component FAME Mix (Sigma Aldrich, Saint Louis, MO, USA). Finally, results were expressed as relative percentage of each fatty acid.

### 2.8. Microbial Challenge Test

Challenge tests were carried out to evaluate the growth potential of *Listeria monocytogenes* LMG 21264, *Escherichia coli* K88, *Salmonella* RISSEN in fermented and unfermented samples, in triplicate. Before use, pathogenic strains were cultured twice, for 24 h at 37 °C, with a 3% *v*/*v* inoculum in TSB added with 0.6% yeast extract (Oxoid, Basingstoke, UK). Afterwards, TSB broth was inoculated (3% *v*/*v*) with each revitalized strain and incubated for 15 h at 37 °C, in order to obtain a cell concentration of 9 Log CFU/mL. Each bacterial culture was then centrifuged (12,857× *g*, 10 min, 4 °C), washed twice in Ringer solution, and suspended in sterile bidistilled water. Puparia, adults, prepupae fermented with both strains and unfermented (control), were inoculated, individually, with each bacterial suspension of pathogen, in order to obtain a final concentration of 6–7 Log CFU/g. The inoculated substrates were incubated at 37 °C and analysed immediately after the inoculum (T_0_), after 24 h (T_24_) and after 48 h (T_48_) of incubation. For microbial counts, 5 g of each sample were homogenized in 45 mL of Ringer solution for 60 s in a Stomacher, and serial 10-fold dilutions were performed. Total viable count was determined using plate count agar on selective medium for each pathogen: Microbiology Chromocult (Merck KGaA, Darmastadt, DE) for the detection of *Salmonella* Rissen and *Escherichia coli* and Listeria selective agar base acc. Ottaviani and Agosti (ALOA) (VWR, Leuven, BE) for *Listeria monocytogenes*.

### 2.9. Statistical Analysis

Data were statistically elaborated by SPSS Statistic 23.0 software (SPSS Inc., Chicago, IL, USA). One-way ANOVA was applied for analysing the results of microbial challenge test, post hoc test Tukey’s HSD were applied to evaluate significant difference (*p* < 0.05) among the means of different samples in different times. Pearson’s correlation between growth ability during fermentation and decrease of pathogenic strains in microbial challenge test was analysed.

## 3. Results

### 3.1. Fermentation

The fermentation of insect waste (puparia and adults insects) and prepupae was carried out by inoculating two different LAB strains, *L. plantarum* (285) and *L. rhamnosus* (1473), at the concentration of 7 Log CFU/g. After 72 h of incubation at the optimal temperature for each species (30 °C for *L. plantarum* and 37 °C *L. rhamnosus*) the strains showed different growth ability (Table 1). *L. plantarum* were able to grow in puparia and adults with an average of 2 Log CFU/g while in prepupae there was an initial decrease from the original inoculum of about 2 Log CFU/g (T_0_). Conversely, *L. rhamnosus* were able to grow only in puparia, with an increase of ca. 2 Log CFU/g, while in prepupae a decrease of about 5 Log CFU/g from initial inoculum was recorded. In adults, although a reduction occurred after inoculum, the growth of *L. rhamnosus* was restored up to 3 Log CFU/g at 72 h.

### 3.2. Black Soldier Fly Composition

In order to investigate the characteristics of the different fermented samples as a function of the different LAB strains used for fermentation, a complete chemical composition analysis of the biomasses obtained from fermentation of BSF prepupae, puparia and dead adults was carried out. The composition in moisture, protein, crude fat, chitin and ash, expressed on wet mass is reported in Table 2.

BSF dead adults and prepupae resulted to be higher in fat respect to puparia. This is explained by the fact that puparia is the protective shell of insects, while the fat accumulation takes place largely inside the insect’s body. Lower amount of lipids are found in BSF prepupae fermented by *L. rhamnosus* respect to the corresponding sample fermented by *L. plantarum*.

Protein content of fermented dead adults and prepupae is higher compared to puparia, in agreement with the data present in the literature [32]. The LAB strain utilized for fermentation does not affect the protein content.

The chitin content, as expected, is higher in fermented puparia samples. Puparia fermented with *L. rhamnosus* and *L. plantarum* respectively reached the levels of 3.9% and 5.2%. In parallel with chitin content, ashes reached the highest level in fermented puparia.

As a whole, the gross composition of the fermented biomasses showed that the main differences can be found on the different insect material used as substrates of fermentation, while only slight effects can be attributed to the different LAB strains used. In order to better understand at a molecular level the composition of fermented samples and to evaluate possible differences between fermented and unfermented samples, fatty acid profile and total amino acid profile were further analysed and compared to the unfermented insect biomasses used as starting material for fermentation.

### 3.3. Variations of Fatty Acid Profile after Fermentation

**Figure 1:**
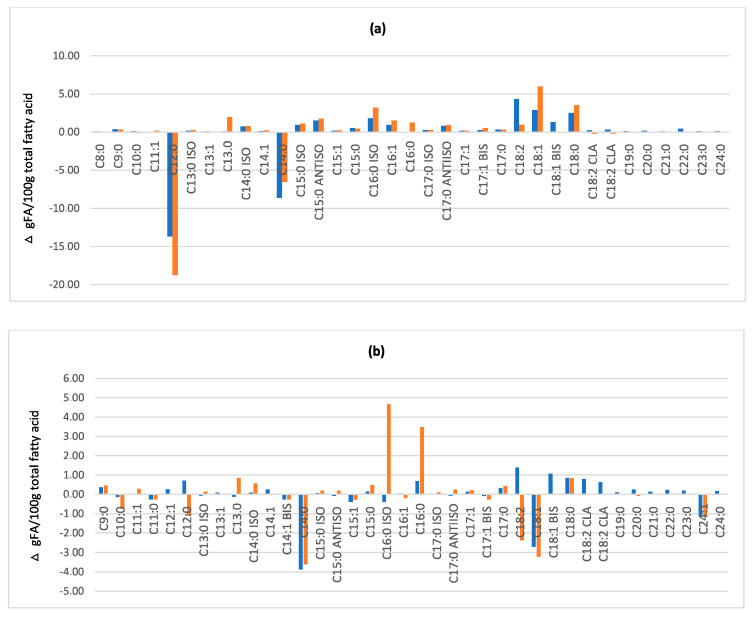

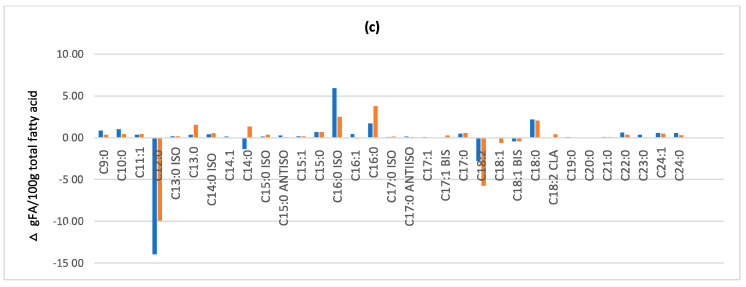
Variations of fatty acids distribution, expressed as relative percentage, in fermented by *L. rhamnosus* (first line)/fermented by *L. plantarum* (second line) BSF prepupae (**a**), puparia (**b**) and dead adults (**c**) compared to corresponding unfermented sample (graph base line).

Fatty acid profile varied considerably after fermentation. The differences between fatty acids distribution in BSF before and after fermentation is shown in Figure 1, with the graph base line representing the starting composition of the unfermented sample, and the bars indicating how much lipid composition changed after fermentation with two different strains. Full data on fatty acid composition is reported in Table S1 of the Supplementary Material. The data showed a consistent fatty acids redistribution. In most cases, there was a reduction of the typical fatty acid of black soldier flies, such as Lauric acid (C12:0) and an increase of minor fatty acids as short chain fatty acids, odd-chain fatty acids, branched chain fatty acids, typical of bacterial cell wall [33].

Therefore, the lipid biomass shifted from a typical composition of insects to a composition containing also fatty acids from lactic acid bacteria metabolism, showing that, in general, the fermentation process modified the insect biomass lipid fraction.

### 3.4. Variations of Total Amino Acid Profile after Fermentation

**Figure 2:**
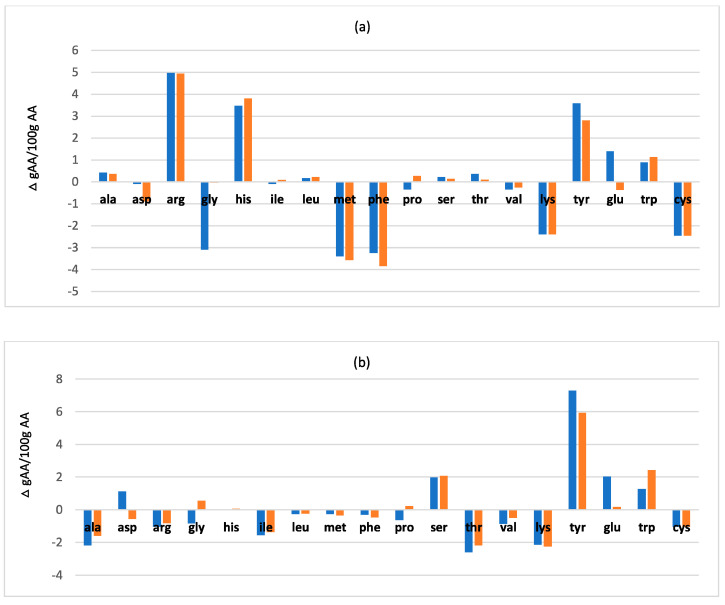

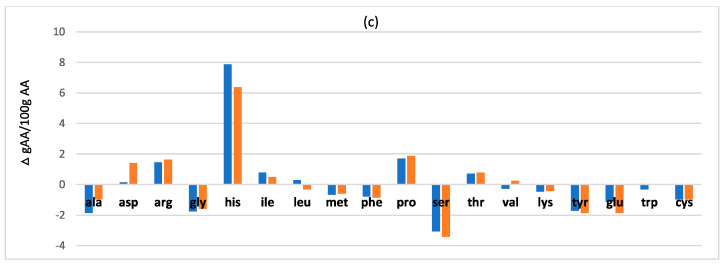
Variations of total Amino Acid distribution, expressed as relative percentage, in fermented by *L. rhamnosus* (first line)/fermented by *L. plantarum* (second line) BSF prepupae (**a**), puparia (**b**) and dead adults (**c**) compared to corresponding unfermented sample (graph base line).

In order to deeply investigate the effect of fermentation on protein fraction, total amino acids profile of the fermented BSF samples were assessed and compared with unfermented samples. Complete data are reported in Table S2 of the Supplementary Material. Figure 2 displays the variation in the total amino acid distribution between BSF fermented and unfermented, expressed in gAA/100 g AA. The graph base line represents the unfermented sample and the bars indicated how total amino acid composition changes for the fermented ones.

The detailed composition of the total amino acids revealed that there was, actually, an amino acids redistribution after fermentation, also depending on the LAB strain, despite the total amount of protein was similar for the two LAB strain used (see Table 2), suggesting that *L. rhamnosus* and *L. plantarum* presented a different protein metabolism.

For the fermented samples of dead adults, the effect of the two LAB strains was very similar, and the most relevant differences before and after fermentation were in the enhanced amount of histidine, proline, arginine, and in the reduction of alanine, glycine, serine, glutamic acid and tyrosine.

In the case of puparia, different changes, common to the both LAB strains, were observed: a reduction of arginine, alanine, isoleucine, lysine, cysteine, threonine. Serine, tyrosine and tryptophan were instead in higher amount respect to unfermented puparia sample. A different behaviour of the two strains was instead observed regarding aspartic and glutamic acid, glycine and proline, both in puparia and prepupae samples. In the samples of prepupae it was observed an higher amount of arginine, tyrosine, serine and tryptophan after fermentation, and a reduction of methionine, phenylalanine, cysteine and lysine.

As a whole, these results suggest that LAB strains used were able to modify the protein composition of the biomass to a different extent, depending both on the specific LAB strain and on the insect biomass.

### 3.5. Antimicrobial Activity

The in vitro antimicrobial activity was carried out by microbial challenge tests, using three pathogenic strains belonging to *L. monocytogenes*, *Salmonella* spp., *E. coli*. The antimicrobial activities of the insect waste (puparia and bodies of dead adult insects) and prepupae were determined by evaluating the growth of different pathogens in fermented and unfermented samples on selective medium for each pathogen.

**Figure 3:**
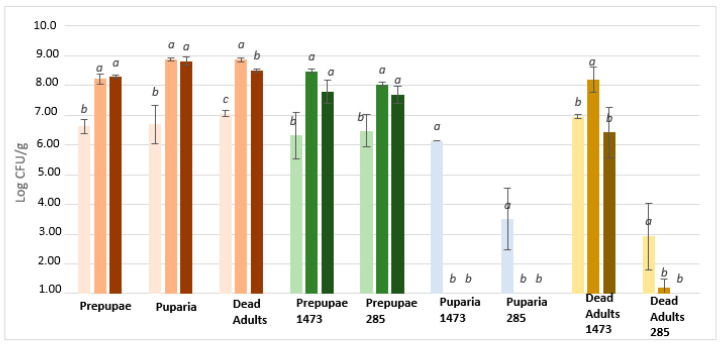
Growth of *Listeria monocytogenes* LMG 21264 on fermented/unfermented insect waste after inoculum (first line/light colour), 24 h (second line/medium colour), and 48 h (third line/dark colour). Starting inoculum 6 Log CFU/g. Letters a-c mark significant (*p* < 0.05) differences among the samples. 1473: fermented with *L. rhamnosus*; 285: fermented with *L. plantarum*.

Unfermented samples inoculated with *Listeria monocytogenes* LMG 21264 (Figure 3) highlight a significant microbial count reduction of about 3 Log CFU/g (*p* < 0.05) after original inocula (ca. 6 Log CFU/g) in prepupae and adults. This trend is not maintained during incubation because in prepupae there was an increase in concentration after 24 h and 48 h, while in adult samples, after an initial decrease there was an increase of 4 Log CFU/g at 48 h. In puparia growth of *Listeria monocytogenes* is recorded after inoculum and after 24 h, while there is a slight decrease after 48 h (ca. 0.90 Log CFU/g) probably due to loss of viability. Moving to all fermented samples, a rapid reduction in total viable cells under the detection limit (1 Log CFU/g) was reached after inoculum (T_0_) and maintained for 48 h of incubation. Interestingly, no correlation was detected between the ability of the strains to grow in insect waste during fermentation and the antimicrobial activity.

**Figure 4:**
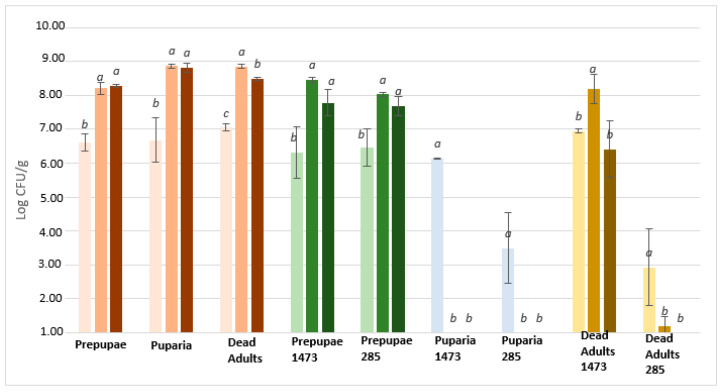
Growth of *Salmonella* Rissen on fermented/unfermented insect waste after inoculum (first line/light colour), 24 h (second line/medium colour), and 48 h (third line/dark colour). Starting inoculum 7 Log CFU/g. Letters a-c mark significant (*p* < 0.05) differences among the samples. 1473: fermented with *L. rhamnosus*; 285: fermented with *L. plantarum*.

Regarding microbial challenge test with *Salmonella* Rissen (Figure 4) a different trend was observed. In this case, unfermented samples didn’t show a decrease in pathogen load. The concentration after inoculum is nearly about the original inocula, ca. 7 Log CFU/g, and an increase of about 1 Log CFU/g was recorded after 24 h.

Differently from the microbial challenge test with *Listeria monocytogenes*, only three of fermented samples shows an antimicrobial activity: puparia fermented with both LAB strains and adults fermented with *L. plantarum*. In particular, a significant reduction (*p* < 0.05) of concentration of pathogens is recorded after inoculum, but a further drop was showed after 24 h until 48 h. On the other hand, fermented prepupae and adults fermented with *L. rhamnosus* revealed similar behaviour to unfermented insects. For these samples a correlation (*r* = 0.69; *p* < 0.05) between microbial counts and antimicrobial activities was detected.

**Figure 5:**
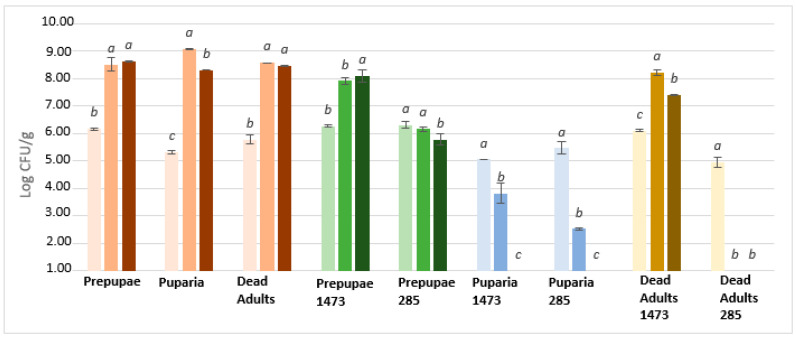
Growth of *Escherichia coli* k88 on fermented/unfermented insect waste after inoculum (first line/light colour), 24 h (second line/medium colour), and 48 h (third line/dark colour). Starting inoculum 7 Log CFU/g. Letters a-c mark significant (*p* < 0.05) differences among the samples. 1473: fermented with *L. rhamnosus*; 285: fermented with *L. plantarum*.

Finally, considering *Escherichia coli* K88, a decrease of its microbial count was observed at T_0_ in all unfermented substrates, but after 24 h and 48 h the growth was restored (Figure 5). Overall, in fermented prepupae and adults fermented with *L. rhamnosus* it was not observed a relevant antimicrobial activity while for the other fermented samples (Puparia 1473, Puparia 285, Dead Adults 285) a trend similar to *Salmonella* was showed.

## 4. Discussion

In the last decades attention is focused to find protein sources alternative to soybean in the formulation of feed diets. This trend is justified giving the fact the world population is predicted to increase and consequently also the food demand. At the same time, the increasing occurrence of outbreaks in livestock caused by pathogenic microorganisms pose the need to discover antimicrobials from natural sources. As fermentation is known to be a strategy to produce metabolites with antimicrobial activity, the rational of this work was to explore the ability of LAB to grow on insect and their wastes looking for possible valorisation of these materials in term of antimicrobial activity. Literature reported that fermentation process is applied during the edible insect production mainly to (i) increase the digestibility and stability of waste prior to the use of insects; (ii) convert food and by product wastes into ingredients of artificial diets for insects (iii) prolong the shelf-life of edible insects [6,24]. Here we report the first study on a potential reuse also of insect waste, focusing on BSF, using fermentation to enhance the antimicrobials properties of raw materials. To reach this purpose, strains of *L. plantarum* and *L. rhamnosus* were used as starter for fermentation of insect waste (puparia and bodies of dead adults) and prepupae. The choice of using these strains is due to the fact that they had recently shown the ability to produce antimicrobial compounds from fermentation of vegetable by-products [23]. Our data demonstrated two main trends: (i) a bacterial growth was observed after LAB inoculum in puparia (ii) a decrease of bacterial count after inoculum was detected and, after 72 h of incubation, a cell growth was restored or a further cell decrease was reached. Thus, interestingly we observed an intrinsic antimicrobial activity of substrates prepupae and dead adults against LAB.

The antimicrobial activity of BSF larvae reared on contaminated substrates or their extracts has been reported in other studies [34,35,36,37,38]. As different authors suggest, the ability of insects to live in extremely harsh environments is favoured by generation of antimicrobial peptides and other substances produced on the surface or within their digestive tract to prevent microbial infection [39].

The key strength of this work is to explore if the antimicrobial activity is present at different stage of the development of BSF and if fermentation can increase it.

Interestingly, after lactic acid fermentation the antimicrobial activity significantly increased.

To note, a strong correlation between the ability to grow and the reduction of pathogens was recorded. Indeed, puparia fermented with *L. plantarum* and *L. rhamnosus* and adults fermented with *L. plantarum* showed the highest LAB growth during fermentation and the highest antimicrobial activity against *Salmonella* Rissen and *Escherichia coli* k88. On the other hand, this trend it is not recorded in microbial challenge test with *Listeria monocytogenes* where all samples showed an antimicrobial activity.

The differences in composition among the three substrates tested could explain the different trend recorded during microbial challenge test. Considering that the main components of insect are protein, fat and chitin, we can presume that during fermentation these substrates were hydrolysed thanks to the enzymatic portfolio of the strains, generating secondary metabolites able to exert the antimicrobial action [40]. The results obtained from molecular analysis actually confirm that insect macromolecules, especially lipids and proteins, have been affected by LAB metabolism. From the bulk composition, fermented adults and prepupae resulted to have highest protein and lipid content, while puparia the highest chitin content. Regarding protein and lipid content, their amounts are lower than those reported in literature for unfermented samples [30,32], suggesting that lipid and protein fractions have been consumed/modified by LAB metabolism. Analysing more in detail both these fractions, a significant redistribution of fatty acid and amino acid profiles was indeed observed. It is known that BSF has a peculiar fatty acid profile [41], being lauric acid (C12:0) the most represented fatty acid in BSF prepupa and adult, whose value can be more than 50% of total detected fatty acids, followed by myristic acid (C14:0) and palmitic acid (C16:0) [30,42]. According to these data, a very similar fatty acid profile was observed in this work in unfermented BSF prepupae and dead adults samples. After fermentation, fatty acid composition of BSF samples was remodelled, moving to a ‘mixed’ profile containing both insects and LAB fatty acids, including minor fatty acids, as branched chain fatty acids, odd chain fatty acids, short chain fatty acids [43,44]. This is in agreement with other studies which showed that fermentation process could significantly change the biomass molecular composition, including fatty acid profile [45,46,47]. The shift in fatty acids profile is common to all the fermented samples, therefore, despite some minor LAB-produced fatty acids can have slight antimicrobial effects, the lipidic fraction alone is not able to explain the different behaviour of fermented samples in the challenge tests.

Total amino acid profile of unfermented BSF samples was also remodelled after fermentation, however it is difficult to find a *rationale* behind the modifications observed, because there is a combined effect of substrate/LAB strain, generating differences among all fermented samples. This is in agreement with other studies showing the ability of LAB to modify the amino acid profile of the fermented biomasses, with differences related to microorganisms and different growth substrate used [48,49,50,51].

In addition, the protein rich sample of dead adults fermented with *L. plantarum*, having the highest antimicrobial activity, showed the same variation in amino acid profile of the adults sample fermented with the other LAB strain with low antimicrobial activity, suggesting that probably the modification of the protein fraction is not the most important factor involved in the antimicrobial effect. However, among the nitrogen fraction, the presence of antimicrobial peptides remains to be explored, eventually produced by the specific LAB strains.

To note, both strains, *L. plantarum* 285 and *L. rhamnosus* 1473, showed the best growth performance and the highest antimicrobial activity when they were grown on puparia. This insect biomass, is represented only by the cuticle that covers insect body and it consists of chitin in different form respect to the prepupae and adults because there is higher sclerotization of the cuticle proteins in the last larval stage. During sclerotization, the insect’s cuticle becomes harder and protect insect during the development and metamorphosis [52]. In fact, as demonstrated by gross composition, puparia contains the highest amount of chitin and minerals and the lower amount of protein and lipid, and this specificity seems to favour LAB growth and the development of antimicrobial properties against pathogens. Although proteolysis, amino acids catabolism and lipolysis are the most studied pathways, LAB have also shown the ability to grow on chitin substrates and hydrolyse it in different derivatives such as chitooligosaccarides and chitosan [53,54]. Despite no specific analysis for the detailed determination of chitin or chitosan oligomers was performed, it is possible that fermentation lead to a modification of chitin with production of antimicrobial compounds. In fact, recently, antibacterial activity of chitosan and its derivatives [55,56,57] has received considerable attention. These molecules have a cationic nature that could interact with negatively charged lipids, proteins, and carbohydrates present on the surface of bacterial cell, inhibiting the transport of solutes and therefore the viability of the pathogens.

The antibacterial effect of chitosan is greater than chitin and depends on the molecular weight and on its deacetylation degree. A preliminary study on genome sequence of the strains used in this work showed the presence of different chitinases (data not shown). This is consistent with other studies arguing that LAB have the ability to express the chitinolytic systems colonization factor [58,59].

## 5. Conclusions

This work aimed to answer questions whether LAB can grow and ferment fractions obtained during rearing of the black soldier fly and whether it is possible to produce high added value molecules during fermentation. As a whole, puparia and adults are fermentable and after fermentation they show antimicrobial activity. The preliminary analysis on LAB fermented insect biomasses showed a shift in lipid and protein composition induced by LAB, suggesting that the fermentation caused important changes in the molecular composition of the biomass analysed. Nevertheless, further studies are necessary to deepen the knowledge about the molecular components responsible for the antimicrobial activity and to highlight the mechanism of action.

## Figures and Tables

**Table 1:** Bacterial counts of two different lactic acid bacteria (LAB) strains in prepupae, puparia and adults of BSF after initial inoculum and after 72 h of fermentation. Data are reported as Log CFU/g (average values ± standard deviation).

	*L. plantarum* 285			*L. rhamnosus* 1473		
	T_0_	T_72_	△ (T_72_–T_0_)	T_0_	T_72_	△ (T_72_–T_0_)
Prepupae	5.61 ± 0.68	7.80 ± 0.57	2.19	4.81 ± 1.30	2.64 ± 0.36	−2.17
Puparia	7.19 ± 0.28	9.36 ± 0.10	2.17	7.15 ± 0.64	9.11 ± 0.29	1.96
Dead Adults	6.65 ± 0.43	8.84 ± 0.48	2.19	4.29 ± 0.81	7.33 ± 0.49	3.04

**Table 2:** Proximate composition of fermented BSF dead adults, puparia and prepupae with two different LAB strains. * Values are expressed on wet matter basis and are the result of four replicate analysis. To be considered for each sample a Sugar content equal to 8.5% added for fermentation process.

Composition (%) *	*L. plantarum 285*			*L. rhamnosus 1473*		
	Prepupae	Puparia	Dead Adults	Prepupae	Puparia	Dead Adults
Moisture (Oven, 105 °C 24 h)	67 ± 0.3	68.4 ± 2.00	76.4 ± 0.40	68.07 ± 0.02	74.0 ± 1.70	76.51 ± 0.05
Lipid (Soxhlet)	5.3 ± 0.5	1.7 ± 0.60	3.85 ± 0.01	3.30 ± 0.90	2.28 ± 0.03	4.30 ± 0.10
Proteins, from total AA (UPLC/ESI-MS)	11 ± 1.00	7 ± 1.00	9.34 ± 0.00	11.15 ± 0.06	6.4 ± 0.30	9.60 ± 0.70
Chitin (UPLC/ESI-MS Determination of Glucosamine)	1.8 ± 0.20	5.2 ± 0.60	1.29 ± 0.09	1.70 ± 0.10	3.9 ± 0.30	1.4 ± 0.10
Ash (Oven 550 °C 5 h + 5 h)	2.07 ± 0.06	5.75 ± 0.02	1.00 ± 0.10	2.00 ± 0.10	4.7 ± 1.10	0.82 ± 0.07

## Supplementary Materials

The following are available online at https://www.mdpi.com/2076-2607/8/11/1785/s1, Table S1: Total Fatty Acid (FA) composition; Table S2: Total Amino Acid (AA) composition.
