# Supplementary material for: Antimicrobial Biomasses from Lactic Acid Fermentation of Black Soldier Fly Prepupae and Related By-Products
Source: Microorganisms. 2020 Nov 14;8(11):1785. doi: 10.3390/microorganisms8111785 (PMC7697071; doi:10.3390/microorganisms8111785)
Supplement: Supplementary file 1 [file microorganisms-08-01785-s001.pdf]

Table S1. Total Fatty acids (FA) compositions of unfermented and fermented BSF dead adults, puparia and prepupae.*All the results are calculated as g FA/100g FA

**FA* (g/100g total fatty acids)**	**Unf D.A.**	**D.A. 1473**	**D.A. 285**	**Unf Pup.**	**Pup. 1473**	**Pup. 285**	**Unf Pre.**	**Pre. 1473**	**Pre. 285**
C8:0	0.00	0.00	0.00	0.00	0.00	0.00	0.00	0.10	0.00
C9:0	0.02	0.86	0.35	0.00	0.37	0.46	0.11	0.48	0.43
C10:0	0.87	1.90	1.32	1.82	1.68	1.08	2.48	2.61	2.37
C11:1	0.00	0.33	0.42	0.00	0.00	0.28	0.00	0.00	0.21
C11:0	0.00	0.11	0.00	0.26	0.00	0.00	0.00	0.00	0.00
C12:0 ISO	0.02	0.00	0.00	0.00	0.00	0.00	0.00	0.04	0.00
C12:1	0.00	0.00	0.00	0.00	0.27	0.00	0.00	0.00	0.00
C12:0	44.05	30.11	34.17	37.24	37.96	36.11	47.09	33.37	28.36
C13:0 ISO	0.11	0.25	0.27	0.22	0.15	0.35	0.00	0.20	0.29
C13:1	0.00	0.00	0.00	0.00	0.09	0.00	0.00	0.07	0.00
C13:0	0.11	0.46	1.68	0.23	0.11	1.08	0.00	0.07	1.96
C14:0 ISO	0.33	0.70	0.85	0.36	0.44	0.91	0.00	0.71	0.79
C14.1	0.24	0.35	0.21	0.20	0.46	0.18	0.16	0.29	0.38
C14:1 BIS	0.00	0.00	0.00	0.26	0.00	0.00	0.00	0.00	0.00
C14:0	12.07	10.69	13.40	14.13	10.26	10.52	15.84	7.25	9.29
C15:0 ISO	0.31	0.44	0.67	0.28	0.36	0.46	0.00	0.94	1.12
C15:0 ANTISO	0.39	0.63	0.44	0.52	0.45	0.71	0.00	1.54	1.77
C15:1	0.07	0.24	0.23	0.52	0.13	0.24	0.00	0.17	0.24
C15:0	0.16	0.80	0.82	0.42	0.57	0.90	0.05	0.60	0.48
C16:0 ISO	0.58	6.52	3.07	1.19	0.81	5.87	0.00	1.84	3.20
C16:1	2.44	2.87	2.32	2.84	2.89	2.67	2.39	3.32	3.92
C16:0	11.50	13.20	15.28	13.71	14.41	17.19	14.26	14.19	15.53
C17:0 ISO	0.10	0.17	0.22	0.12	0.14	0.23	0.00	0.30	0.30
C17:0 ANTIISO	0.14	0.25	0.22	0.26	0.19	0.50	0.00	0.85	0.94
C17:1	0.11	0.18	0.11	0.13	0.27	0.33	0.00	0.19	0.17
C17:1 BIS	0.29	0.29	0.54	0.27	0.18	0.00	0.00	0.29	0.51
C17:0	0.15	0.63	0.71	0.32	0.65	0.75	0.08	0.44	0.44
C18:2	12.09	9.33	6.33	8.05	9.45	5.66	8.93	13.27	9.92
C18:1	10.37	10.34	9.77	11.72	9.00	8.49	6.79	9.71	12.75

C18:1 BIS	0.42	0.00	0.00	0.00	1.06	0.00	0.00	1.32	0.00
C18:0	2.19	4.36	4.26	3.60	4.44	4.43	0.96	3.48	4.52
C18:2 CLA	0.00	0.00	0.40	0.00	0.79	0.00	0.20	0.43	0.00
C18:2 CLA	0.00	0.00	0.00	0.00	0.63	0.00	0.20	0.54	0.00
C19:0	0.01	0.08	0.05	0.00	0.11	0.00	0.00	0.13	0.00
C20:0	0.12	0.13	0.09	0.09	0.32	0.00	0.01	0.18	0.03
C21:0	0.00	0.09	0.07	0.00	0.13	0.00	0.00	0.09	0.00
C22:0	0.09	0.70	0.43	0.35	0.57	0.38	0.01	0.43	0.00
C23:0	0.00	0.32	0.00	0.00	0.20	0.00	0.00	0.15	0.00
C24:1	0.00	0.57	0.47	1.10	0.00	0.00	0.00	0.00	0.00
C24:0	0.08	0.66	0.39	0.00	0.17	0.00	0.00	0.13	0.00

Unf. D.A. unfermented dead adults; D.A.1473 dead adults fermented with *L. rhamnosus* 1473; D.A. 285 dead adults fermented with *L. plantarum* 285; Unf. Pup. unfermented puparia; Pup.1473 puparia fermented with *L. rhamnosus* 1473; Pup. 285 puparia fermented with *L. plantarum* 285; Unf. Pre. unfermented prepupae; Pre.1473 prepupae fermented with *L. rhamnosus* 1473; Pre. 285 prepupae fermented with *L. plantarum* 285.

Table S2. Total Amino Acid (AA) composition of unfermented and fermented BSF dead adults, puparia and prepupae. *All the results are calculated by dry mass as gAA/100g AA and expressed as means ± standard deviation of two replicate analysis.

**AA (g/100g aa)***	**Unf. D.A.**	**D.A. 1473**	**D.A. 285**	**Unf. Pup.**	**Pup. 1473**	**Pup. 285**	**Unf. Pre.**	**Pre. 1473**	**Pre. 285**
ala	9.7±0.9	7.84±0.06	8.7±0.3	11.7±0.5	9.5±0.2	10.1±0.6	5.5±0.3	5.9±0.1	5.9±0.1
asp	10.2±1.1	10.3±1.7	11.65±0.09	7.9±0.5	9.0±0.2	7.3±0.4	8.2±0.4	8.1±0.8	7.34±1.03
arg	6.7±0.8	8.2±0.5	8.3±0.5	3.7±0.4	2.7±0.1	2.95±0.05	4.89±0.03	9.8±0.1	9.8±1.1
gly	6.8±0.4	5.07±0.05	5.23±0.07	13.8±0.7	12.9±0.2	14±1	6.01±0.01	2.9±3.5	5.9±0.1
his	0.002±0.001	7.8±2.09	6.35±0.06	0.12±0.02	0.16±0.02	0.1±0.1	3.56±0.04	7.04±0.09	7.3±0.6
ile	5.25±0.02	6.0±0.6	5.7±0.3	3.94±0.09	2.3±0.2	2.5±0.1	4.00±0.09	3.9±0.1	4.0±0.2
leu	7.9±0.1	8.27±0.01	7.6±0.2	7.3±0.1	7.1±0.3	7.1±0.1	6.9±0.1	7.0±0.1	7.11±0.07
met	2.2±0.2	1.5±0.2	1.6±0.4	0.50±0.01	0.24±0.01	0.15±0.03	6.45±0.04	3.05±0.19	2.89±0.07
phe	3.7±0.3	2.9±0.2	2.8±0.4	2.2±0.1	1.89±0.06	1.72±0.09	11.5±0.2	8.3±1.3	7.7±0.5
pro	2.8±3.2	4.5±0.1	4.7±0.5	7.6±0.1	7.016±0.009	7.8±0.2	5.78±0.09	5.4±0.2	6.05±0.46
ser	5.080±0.003	2.0±0.3	1.6±0.3	6.2±0.1	8.2±0.5	8.3±0.3	4.23±0.08	4±1	4.35±0.09
thr	4.8±0.1	5.5±0.2	5.6±0.5	3.6±0.1	1.0±0.3	1.4±0.3	3.78±0.02	4.1±0.6	3.8±0.5
val	6.6±0.1	6.3±0.3	6.8±0.4	7.8±0.1	7.0±0.4	7.3±0.4	6.2±0.1	5.8±0.2	5.9±0.1

lys	7.7±1.5	7.2±0.6	7.3±0.4	3.2±0.4	1.1±0.1	1.0±0.1	4.6±0.2	2.2±0.8	2.27±0.07
tyr	4.1±0.7	2.4±0.2	2.3±0.3	5.1±0.3	12.4±0.5	11.0±0.5	6.2±0.2	9.8±1.9	9.0±0.7
glu	13.2±1.8	12.1±0.6	11.37±0.02	10.7±0.6	12.7±0.3	10.8±0.6	8.9±0.5	10.2±0.8	8.5±1.7
trp	1.6±0.1	1.35±0.06	1.68±0.08	2.9±0.1	4.2±0.2	5.3±0.6	0.46±0.03	1.36±0.05	1.6±0.1
cys	0.9±0.7	-	-	1.05±0.08	-	-	2.4±0.1	-	-

Unf. D.A. unfermented dead adults; D.A.1473 dead adults fermented with *L. rhamnosus* 1473; D.A. 285 dead adults fermented with *L. plantarum* 285; Unf. Pup. unfermented puparia; Pup.1473 puparia fermented with *L. rhamnosus* 1473; Pup. 285 puparia fermented with *L. plantarum* 285; Unf. Pre. unfermented prepupae; Pre.1473 prepupae fermented with *L. rhamnosus* 1473; Pre. 285 prepupae fermented with *L. plantarum* 285.
